# Examination of new clinical dental caries in school children using real intra oral photos with artificial intelligence model YOLO-V8x

**DOI:** 10.1186/s12903-025-07486-x

**Published:** 2025-12-20

**Authors:** Rina Putri Noer Fadilah, Rasmi Rikmasari, Saiful Akbar, Arlette Suzy Setiawan

**Affiliations:** 1 Doctoral Program, Faculty of Dentistry, Padjadjaran University, Bandung, Indonesia; 2 Departement of Dental Public Health, Faculty of Dentistry, Jenderal Achmad Yani University, Cimahi, Indonesia; 3 Department of Pediatric Dentistry, Faculty of Dentistry, Padjadjaran University, Bandung, Indonesia; 4 School of Electrical Engineering and Informatics, Institute Technology of Bandung, Bandung, Indonesia; 5 Departement. of Prosthodontic Dentistry, Faculty of Dentistry, Padjadjaran University, Bandung, Indonesia

**Keywords:** Dental caries, ICDAS, School children, YOLO-V8x

## Abstract

**Background:**

Dental caries is a common chronic condition among school children, and in Indonesia, rates have been higher than 80%. Traditional diagnostic techniques tend to be long and dependent upon the availability of healthcare professionals and hence promote development of alternatives involving artificial intelligence (AI). This work aimed to evaluate the effectiveness of the application titled HI Bogi that embeds the model known as YOLOv8 in distinguishing dental caries among primary school pupils in Cimahi, Indonesia.

**Materials and methods:**

A cross-sectional analytic model with prospectively collected data was adopted. A dataset consisting of 3,221 JPG photographs was created and labeled based on the International Caries Detection and Assessment System (ICDAS, D0 - D6) using Roboflow software. The photographs were resized to a size of 640 × 640 pixels and distributed into training (2,266 photographs), validation (635 photographs), and testing (320 photographs) sets. A YOLOv8x algorithm was used for application in deep learning tasks, and its performance was determined through mean Average Precision (mAP), Intersection over Union (IoU) measurements, and precision and recall metrics. Additionally, a Mann Whitney statistical test was carried out to contrast classification efficiency between the AI system and dental practitioners’ classification efficacy, while both methods’ diagnostic speed was tested.

**Results:**

mAP was 45.8%, and precision was 72.6%, and recall was 41.1% for YOLOv8x model. Comparative testing across ICDAS categories did not vary significantly between dentists and AI (*p* > 0.05). Detection of caries (D1–D6) was observed to have a sensitivity ranged from 82.31% to 96.45% and specificity ranged from 77.10% to 99.33% on new data. Additionally, testing was completed approximately four times faster with AI compared to manual testing (*p* = 0.000).

**Conclusion:**

The YOLOv8x model in the HI Bogi app demonstrated diagnostic performance equal to dentists and a drastically shortened examination time, validating its use in school-based dental health programs. But class-specific precision variation suggests further refinement is needed. Further studies ought to increase datasets and characterize advanced architecture, such as transformer-based architecture, to achieve higher specificity and detection rates in rare lesion categories.

## Background

Dental caries, commonly known as tooth decay, is one of the most prevalent chronic diseases in school-aged children worldwide. Based on Indonesian survey, the prevalence of dental caries among children is more than 80% [1]. Despite significant advancements in dental care, early detection and effective intervention remain critical to prevent severe dental complications and improve oral health outcomes. Traditional diagnostic methods often rely on clinical examinations, which, although effective, can be time-consuming and require highly trained professionals [2]. Therefore, early detection of caries is crucial to reduce treatment costs and time, as well as to prevent further complications. In this context, the development of more detailed and equitable school-based dental health programs across Indonesia is urgently needed to prevent oral diseases, including caries. One of the programs that has long been implemented is the school dental health program (*Usaha Kesehatan Gigi Sekolah* or UKGS). Although it has novel objectives, the implementation of this program has not been optimal, leaving many students without adequate dental health services.

One of the main challenges in screening caries status in elementary schools is the limited time and number of healthcare professionals, such as dentists and dental nurses, at community health centers (*Puskesmas*). To address this issue, the HI Bogi application was developed, enabling independent caries status screening through mobile devices. However, this application still requires results interpretation by dentists, which takes a considerable amount of time, thus posing challenges to its efficiency [3, 4].

As a solution, the application of Artificial Intelligence (AI) based on the The Internet of Dental Things (IoDT) refers to applying IoT (Internet of Things) concepts specifically to dentistry. In practice this means dental devices, sensors, and equipment are connected via networks (often cloud-based) to collect and share oral-health data. Researchers describe IoDT as an “innovative approach to achieve prevention and management of dental caries, periodontal diseases, oral cancers, and other dental diseases [5]. AI can mimic human thinking processes, allowing caries status assessments to be performed in real time and efficiently. Recent advancements in artificial intelligence (AI) have opened new avenues for enhancing the diagnostic accuracy and efficiency of dentistry. This technology offers the potential for early caries detection, prevention of more severe dental damage, and improvement of the effectiveness of screening in elementary schools. Therefore, research on integrating AI technology into the HI Bogi application needs to be conducted. This study aims to create a system that not only accelerates the caries status assessment process but also ensures that AI assessment results are equivalent to those of dentists, particularly based on the International Caries Detection and Assessment System (ICDAS) classification. Additionally, this research is essential to evaluate the time efficiency of AI-based and conventional methods in caries assessment processes. This integration is expected to be a strategic step in enhancing the effectiveness and efficiency of caries screening programs for elementary school children in Indonesia [3, 4, 6, 7].

Among these advancements, object detection models such as You Only Look Once (YOLO) have demonstrated remarkable potential for analyzing medical and dental images. YOLO-v8, as the latest iteration of this model, offers improved accuracy and speed, making it particularly suitable for real-time diagnostic applications [8, 9]. More recent research using the YOLOv5x model achieved respectable performance metrics precision of 0.786, recall of 0.618, and F1-score of 0.692 in detecting white spot lesions [10], while the YOLOv5l model reported even higher precision of up to 0.853 [11]. However, many of these studies relied on controlled datasets with limited population diversity and often lacked integration into real-world, community-based dental settings.

In this study, we used real intraoral photographs to examine the utility of YOLO-v8 in detecting dental caries among schoolchildren. This approach combines the robustness of AI-powered diagnostics with practical, noninvasive image acquisition techniques, potentially transforming the traditional caries-detection paradigm. Our investigation aimed to evaluate the performance of this model, highlight its practical applications, and explore its implications for school-based dental health programs [8–10, 12].

## Materials and methods

### Ethical approval and consent to participate

This study was conducted in accordance with the ethical standards of the Declaration of Helsinki (1964, and its later amendments) and received ethical approval from the Research Ethics Committee of the Faculty of Dentistry, Padjadjaran University (approval number: 1291/UN6.KEP/EC/2023; registration number: 2309051343; Clinical trial number: not applicable). Written informed consent to participate was obtained from the parents or legal guardians of all participating children under the age of 16 prior to data collection.

### Study design

This study employed a cross-sectional diagnostic design with prospective data collection using intraoral clinical photographs collected by multiple research teams to train the model. The standard for classifying caries in this study used a classification based on the ICDAS six stages of caries [13]. We then trained the YOLO-V8 deep learning model to classify dental caries and evaluated the training results using the data split collected in Table 1. Then, we conducted a direct field retest evaluation with dentists using the criteria of sensitivity, specificity, and a comparison of the time taken to examine dental caries by dentists and artificial intelligence with the total data tested in Table 4. This study referred to the STARD standards for reporting diagnostic accuracy [14].

**Table 1:** Distribution of labels on datasets

Labeled	Number of Instances
D0	1000
D1	900
D2	1200
D3	1100
D4	900
D5	1300
D6	1400

### Datasets

In a study collecting the data needed to compile the dataset, researchers found that there are differences in dental characteristics between Indonesian residents and residents of various countries [15]. Therefore, we examined the differences between the characteristics of data collected primarily from Indonesians, which we have presented in previous studies IDCCD [16].

### Research team preparation and calibration

Before going into the field for data collection and annotation, the research team underwent a structured calibration program to ensure diagnostic consistency. The team consists of eight dentists and young dental practitioners, supported by four dental students as technical assistants, and eight dental students who took pictures. The calibration process includes theoretical and practical training sessions based on the International Caries Detection and Assessment System (ICDAS) protocol, as described in our previous publication [17]. The calibration involved only the eight dental students. Inter-rater reliability among the eight dental students, computed using Cohen’s kappa, ranged from 0.61 to 0.90, indicating substantial to almost perfect agreement.

### Data collection sites and imaging technique

Primary data in this study were collected by eight dental students as photographers who had been trained in calibration and eight dentists and young dentists as supervisors. The data was collected directly from several locations, including the North Cimahi Community Health Center, Central Cimahi Community Health Center, South Cimahi Community Health Center, Mandiri 02 State Elementary School, Mandiri 04 State Elementary School, Mandiri Baros Cimahi State Elementary School, Mandiri Melong 04 State Elementary School, and Cibabat 2 State Elementary School. Data were collected from children aged 6–12 years, and data selection used convenience sampling. Intraoral images were taken using mobile devices, including iPads and smartphones with a minimum camera resolution of 12 megapixels. Images were taken in dry field conditions with standard lighting, in accordance with the data collection protocol from the IDCCD study [15]. To guarantee the completeness and readability of visual information for the detection of caries, images were captured from several standardized angles. These were frontal for the sake of capturing anterior teeth, right and left laterals for posterior areas, occlusal to capture maxillary (upper) arches, and sub-occlusal to capture mandibular (lower) arches. This multi-perspective imaging approach was intended to bring out dental caries in different surfaces, including occlusal, proximal, and cervical surfaces, which are usually difficult to detect using single-view photographs. This yielded a dataset of 3,221 clinical intraoral photographs.

### Annotation process and dataset composition

Following image acquisition, all intraoral photographs were processed through a structured, multi-phase annotation workflow to ensure high-quality and consistent labeling. Initially, Eight qualified dentists and young dentists performed initial annotation by identifying and labeling each tooth surface based on the International Caries Detection and Assessment System (ICDAS), ranging from code D0 (sound tooth) to D6 (extensive cavitation). The labeled images were then imported into a semi-automated annotation tool, Roboflow, that enabled accurate bounding box placement to align with annotated ICDAS labels. To ensure the accuracy of the diagnosis, two qualified ICDAS experts verified the annotation results independently. Only the images on which both experts agreed were included in the final dataset, which maintained consistency and minimized personal bias in the labeling. The final dataset had a good combination for all ICDAS categories as shown in Table 1.

### Classification

The classification of the collected data focused on labeling the data in the dental caries class using guidelines from the Index of Dental Caries and Early Detection (ICDAS) [13, 18]. The International Caries Detection and Assessment System (ICDAS) classifies dental caries into seven distinct stages, ranging from D0 to D6 [19], which reflect the progression of carious lesions from non-cavitated to cavitated forms. Stage D0 indicated a sound tooth surface with no evidence of caries. In contrast, stages D1 and D2 represent early carious lesions, where D1 corresponds to non-cavitated lesions that are visible only under specific conditions, such as drying of the tooth surface, whereas D2 denotes non-cavitated lesions that are visible in a wet environment [20]. Stages D3 to D6 signify progressively more severe carious lesions, with D3 indicating a cavitated lesion confined to enamel, D4 representing a lesion that has progressed into dentin but does not yet affect the pulp, and D5 and D6 indicating advanced carious lesions with significant dentin involvement, where D5 is characterized by extensive cavitation and D6 reflects severe carious lesions that may involve the pulp [21]. This classification system not only aids in the diagnosis and management of caries but also facilitates communication among dental professionals regarding the severity and treatment needs of carious lesions [22]. The ICDAS framework has been widely adopted in clinical practice and research, providing a standardized approach to caries assessment that enhances the accuracy of diagnosis and treatment planning [23]. The following are samples of intraoral clinical photographs labeled by the researchers according to the ICDAS classification in Table 2.

**Table 2:** Class label classification guide on datasets with ICDAS method

No	Data images	Class/Caries Stage	Caries Extension
1		D0	No clinically detectable lesion. Dental hard tissue appears normal in color, translucency, and gloss.
2		D1	Earliest clinically detectable lesion compatible with mild demineralization. Lesion limited to enamel or to shallow demineralization of cementum/dentin. Mildest forms are detectable only after drying. When established and active, lesions may be white or brown and enamel has lost its normal gloss.
3		D2	
4		D3	Visible signs of enamel breakdown or signs the dentin is moderately demineralized.
5		D4	
6		D5	Enamel is fully cavitated and dentin is exposed. Dentin lesion is deeply/severely demineralized
7		D6	

### The model

The methodology for utilizing YOLOv8 in the detection of dental caries from imaging data is grounded in its advanced architecture and training techniques, which collectively enhance its performance in real-time applications [24]. YOLOv8 was built on the CSPDarknet53 backbone, which is an evolution of the traditional Darknet architecture that integrates cross-stage partial (CSP) networks. This design significantly augments the model’s learning capacity and efficiency, allowing for improved feature extraction from dental images [25]. The architecture comprises three primary components: backbone, neck, and head. The CSPDarknet53 backbone is responsible for extracting hierarchical features, thereby creating a comprehensive visual representation of the input image [26, 27]. The path-aggregation network (PANet) serves as a neck that facilitates effective feature fusion across multiple scales, which is crucial for accurately detecting objects of varying sizes, including early carious lesions [18]. The YOLO head is tasked with generating bounding box predictions, objectness scores, and class probabilities, which are essential for identifying the presence of caries in dental images [28]. Furthermore, YOLOv8 employs advanced training methodologies, such as Rectified Adam (RAdam) optimization and data augmentation techniques like MixUp, which contribute to faster convergence and enhanced generalization during the training process [29]. These improvements render YOLOv8 highly adaptable to diverse datasets and application contexts, ensuring its efficacy in various computer vision tasks, including real-time object detection and segmentation, which are particularly relevant for the early detection of dental caries [30].

### Training and test of the model AI

In the training phase of this study, the Google Colab Pro platform equipped with an A100 GPU and 54GB RAM was used. This platform was chosen because of its superior ability to handle computationally intensive deep learning tasks. In training the deep learning model in this study using a deep learning algorithm or model, namely YOLO - V8x with training using datasets that have been built by researchers show data with ground truth referring to appropriate references and have been validated by experts in the field of dentistry, namely dentists. The Ground truth represents the targeted result in the model learning process and is used to support in the model learning process and is used to support model training, evaluate its performance and report results [31]. In this study, the Non-maximum Suppression (NMS) method is used for cases where more than one segmentation is performed. In cases with multiple detection results such as bounding boxes, it was used to filter these results and select the best result [32]. In this algorithm there are parameters set to produce accurate detection objects with 50% overlap confidence that will eliminate objects with probability lower than 50% so that the detected results are close to the ground truth [33].

The model was trained using a dataset of 640 × 640 pixel images, comprising 3,221 images divided into three groups: 2,266 images for training, 320 images for testing, and 635 images for validation. This division is crucial for ensuring that the model can generalize well to unseen data, as highlighted in recent literature emphasizing the importance of proper dataset partitioning in machine learning tasks [34]. The training process lasted for 300 epochs, which was expected to provide the model with sufficient time to learn the underlying patterns in the data comprehensively [35].

The optimizer employed during this training was AdamW, which is a variant of the Adam optimization algorithm that incorporates weight decay to mitigate overfitting [36]. The learning rate was set to 0.000909, and the momentum was set to 0.9. These hyperparameters are critical for the convergence and stability of the training process, as discussed in various studies that have explored the impact of learning rates and momentum on model performance [37, 38]. The parameter optimizer was categorized into three distinct groups to address the varying regularization needs of different parameter types. The first group contained 97 weight parameters without weight decay (decay = 0.0), which are typically reserved for parameters that do not require additional regulation [39]. The second group included 104 weight parameters with a weight decay of 0.0005, serving as a regulatory mechanism to reduce model complexity and prevent overfitting, which is a common challenge in deep learning [40]. The third group comprised 103 bias parameters that were not subjected to weight decay (decay = 0.0), consistent with practices aimed at optimizing the model training [41]. This parameter division is performed to ensure that each parameter group receives the appropriate treatment so that the training process can run optimally, and the resulting model has good generalization to data that has never been seen before.

**Fig. 1:**
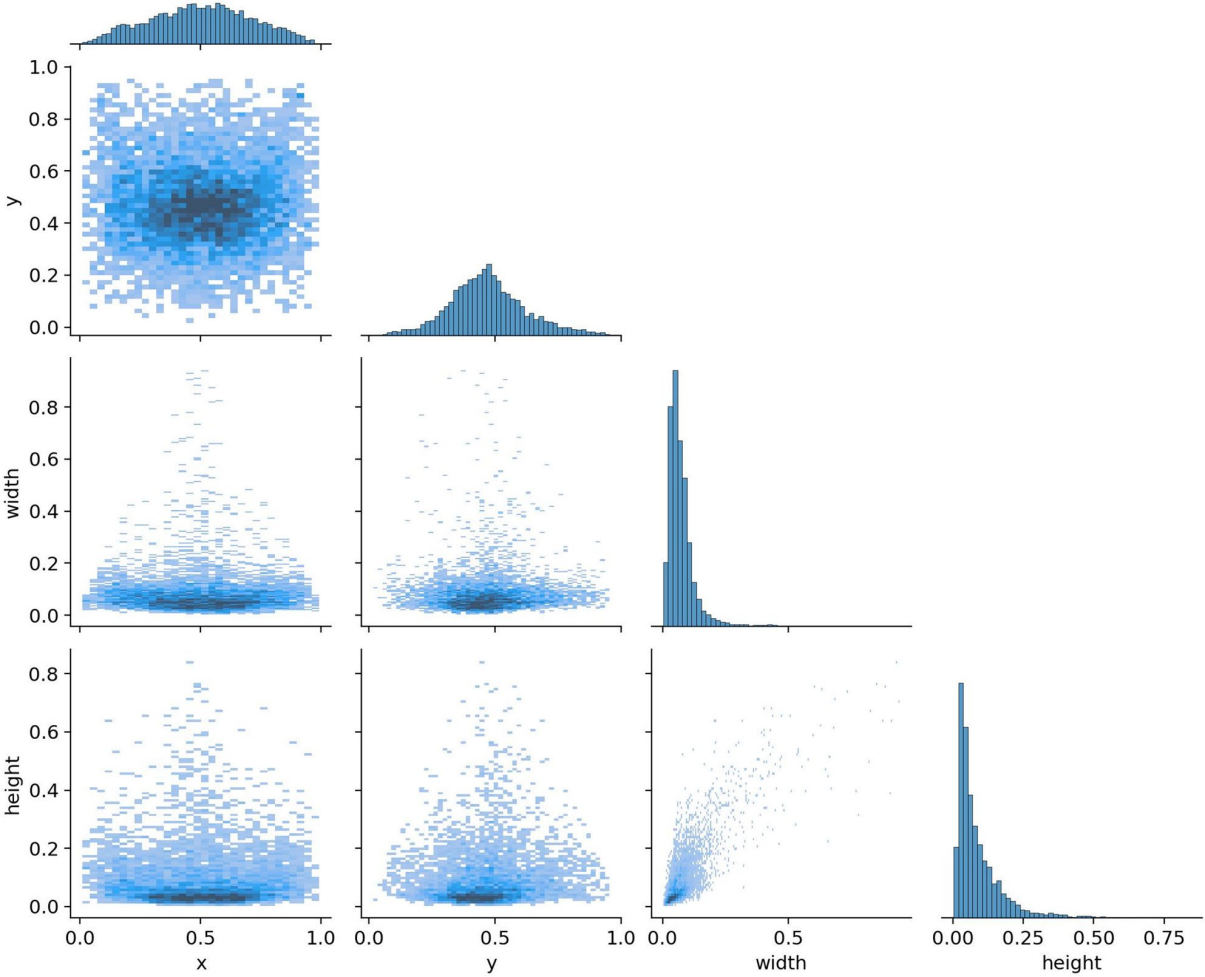
Correlogram showing YOLOv8x performance

After the training, the best model is saved. The performance of YOLOv8x is shown in the correlation in Fig. 1 shows a correlogram illustrating the distribution and relationship between the bounding box coordinates (x, y) and sizes (width, height) of caries detected in the training dataset used by the YOLOv8x model. Each part of the graphical illustration clarifies the relationships among these variables. For instance, the topmost row and the leftmost column outline the common areas where caries occur in the image mostly located towards the center (with x and y values close to 0.5). The graphs also show that most detected caries are of smaller sizes, as supported by the limited distributions of width and height. The presence of correlation between width and height suggests that larger caries would more likely have higher height and width, which is consistent with expected results. This correlogram is helpful in improving our understanding of the data used to train the artificial intelligence model, thus enhancing the fact that the distribution of object locations and sizes represents real-world situations and supports the model’s efficiency in caries detection.

### Statistical analysis

For model evaluation, several metrics essential for assessing object detection performance were used. These metrics include Intersection over Union (IoU), mean Average Precision (mAP), and Average Precision (AP) [24]. The IoU measures the extent to which the bounding box prediction of the model overlaps with the ground truth using the mathematical equation in Eq. (1) [12]. mAP, which is the average AP for different object classes, provides an overall picture of the detection accuracy of the model on the dataset used with the mathematical equation in Eq. (2) [42]. The AP itself is a metric that evaluates the precision of the model for each class at various IoU threshold levels, providing a more detailed view of the model’s performance in detecting each object type using the mathematical equation in Eq. (3).

1 $$\:IoU=\:\frac{(groundTruth\cap\:prediction)}{(groundTruth\cup\:prediction)}$$

2 $$\:mAP=\:\frac{1}{n}{\sum\:}_{i=1}^{n}{AP}_{i}$$

3 $$\:AP={\sum\:}_{k=0}^{K}\left({R}_{r}\left(k\right)-{R}_{r}\left(k+1\right)\right){Rr}_{interp}\left({R}_{r}\left(k\right)\right)$$

4 $$\:Precision=\:\frac{TP}{TP+FP}\:$$

5 $$\:Recall=\frac{TP}{TP+FN}$$

Where:

TP: True positive when the model correctly predicts a positive sample.

FP: false positive; when the model predicts a positive sample, it is negative.

FN: False negative; when the model predicts a negative sample, it is positive.

To statistically support the evaluation of artificial intelligence system performance, formal sample size calculations were performed. The total sample size was determined using a formula designed to compare the proportions between two groups:

6 $$\:N=\:\frac{\left(\frac{1}{q1}+\frac{1}{q2}\right)\:{S}^{2}{\left({Z}_{a}+{Z}_{b}\right)}^{2}}{{E}^{2}}$$

In this Eq. (6), $\:{S}^{2}$ refers to the population variance, $\:{Z}_{a}$=1.96 corresponds to a 5% significance level (α = 0.05), and $\:{Z}_{b}\:$=0.84 reflects an 80% power level (β = 0.20). $\:q1$​ and $\:q2$​ denote the proportions of subjects in each comparison group, while $\:E$ = 0.2 represents the acceptable margin of error. Based on this calculation, the minimum required number of study participants was found to be 394. This ensured sufficient statistical power to detect meaningful differences in diagnostic performance between the AI model and traditional methods when applied to the intraoral images of school-aged children.

To compare the sensitivity and specificity of dentists with the AI model within each ICDAS classification, a two-proportion Z-test was employed since the data were categorical proportions, individual *p*-values were calculated for every classification [43]. In parallel, to compare the diagnostic accuracy and examination time, a bivariate analysis using the Mann-Whitney U test was used to examine the differences between the performance of the AI system and the direct clinical examinations done by the dentists in terms of the assessment of dental caries among elementary school children [44].

## Results

The results of this study indicate that the YOLO-V8x model trained using the dataset achieves satisfactory performance. As shown in Table 3, the model achieves an average precision ranging from 33% to 66% across various ICDAS categories. The average precision (mAP) at 50% Intersection Over Union (IoU) is 45.8%, with a precision of 72.6% and a recall of 41.1%.

**Table 3:** YOLO-V8x model training results report with testing using test datasets

ICDAS Classification	Average Precision	Mean average precision (mAP)	Intersection Over Union (IOU)	Precision	Recall
D0	39%	45,8%	50%	72,6%	41,1%
D1	38%				
D2	41%				
D3	33%				
D4	39%				
D5	61%				
D6	66%				

Additional testing with new data conducted directly in the field at a different time and place from the data used in training but still in the Cimahi area, as shown in Table 4, also yields consistent results. Prediction labels align with labeled data, with an average model confidence above 80%, as shown in Fig. 3.

**Table 4:** New test data collected directly in the field with the frequency of labels for each stage of caries in cimahi

ICDAS Classification	Frequency	Percentage (%)
D1	201	13,52
D2	270	18,16
D3	222	14,93
D4	249	16,75
D5	267	17,96
D6	277	18,64
Amount	1486	100

To evaluate the model’s diagnostic competence, a comparison with an experienced dentist was conducted on 394 field-collected test data, with the ICDAS category distribution related to this evaluation shown in Table 4. As shown in Table 5, statistical analysis indicates no significant difference in performance between the AI model and dentists across all ICDAS categories, with p-values ranging from 0.301 to 0.693 (all *p* > 0.05). This indicates that both the AI model and dentists have comparable performance levels in terms of sensitivity, specificity, positive predictive value (PPV), and negative predictive value (NPV).

**Table 5:** Data analysis of the comparative results between the AI model and dentists

ICDAS	Dentists				Artificial Intelligence				*P* value
	SEN	SPE	PPV	NPV	SEN	SPE	PPV	NPV	
D1	89,80	90,56	80,83	95,29	90,80	90,17	82,57	95,04	0,301
D2	82,84	84,11	80,45	86,12	82,31	84,47	79,88	86,41	0,690
D3	83,53	71,33	85,59	73,2	83,12	77,10	85,23	74,21	0,621
D4	86,41	84,29	84,12	86,55	86,77	83,87	84,53	86,18	0,693
D5	96,17	99,31	99,43	95,36	96,45	99,33	99,45	95,45	0,634
D6	95,20	90,00	87,42	96,27	95,59	89,36	88,37	96,28	0,302

Additionally, examination time was compared between the AI model and dentists. The AI model demonstrated significantly shorter diagnostic procedure times (mean = 31.95 ± 3.2 s) compared to dentists (mean = 133.61 ± 6.53 s), with a p-value of 0.000, indicating a statistically significant difference. These results indicate that not only does the AI model offer diagnostic accuracy comparable to that of expert dentists, but it also provides a significantly faster option, which could enhance the efficiency of caries diagnosis in clinical applications.

## Discussion

Assessing caries in teeth, especially in the early stages of caries, such as white spot lesions, is quite challenging. There are several ways to assess caries in teeth, one of which is with intra oral photographs [45] or with radiographs. Utilizing artificial intelligence technology is quite helpful in assessing early stage caries in teeth with deep learning [46]. In previous studies, satisfactory results have been found in detecting caries with intelligent systems or AI with the YOLO v3 and Faster R-CNN models [12]. In addition, in detecting caries in realtime, good results were obtained using the YOLO v5x model with the results of performance metrics including precision, recall, and F1 score values for detecting white spot lesions are 0.786, 0.618, and 0.692 [10]. In subsequent research using the YOLO v5 model showed even better results where the highest precision (0.853) occurred in the YOLOv5l model [11]. In addition, our previous research investigated which model was best suited for the dataset in this study, and found that among the three AI models Faster-RCC, YOLO v8, and DETR, YOLO v8 with architecture x yielded the best results, with a MaP of 41.8 and an IoU of 50% [16].

**Fig. 2:**
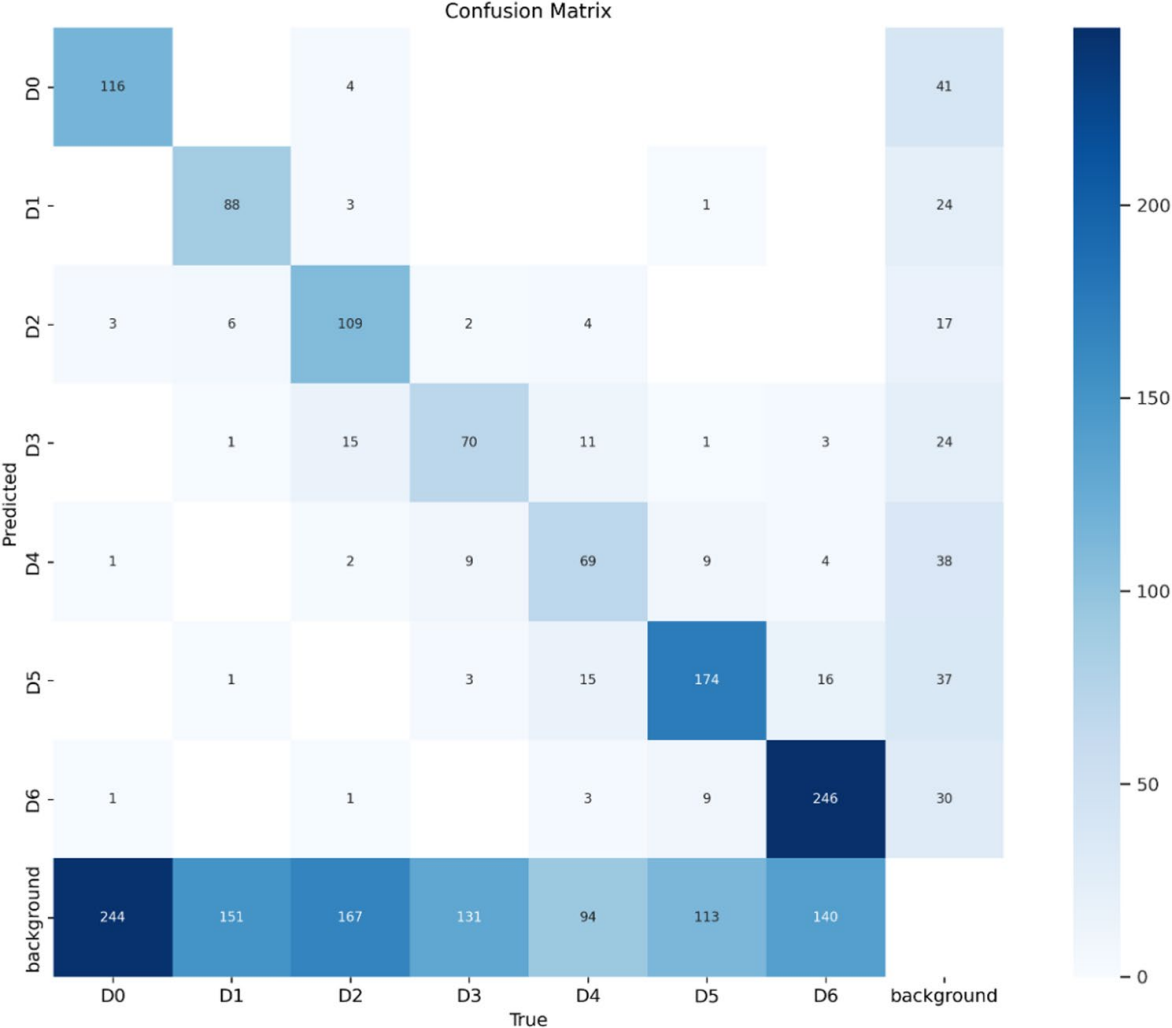
Confusion matrix

In this study, we found a gap supported by previous research related to the development of AI models for caries stage assessment. Previous research has not used a caries assessment approach with a classification based on the International Caries Detection and Assessment System (ICDAS), which divides caries into seven stages ranging from no caries (D0) to advanced stage (D6). The results of training a dataset with seven caries classes using the YOLO v8x deep learning model showed good results, However, the variation in precision and recall between classes indicates that the model is not consistent in detecting all classes with the same accuracy, with the model sometimes not classifying the label but identifying it as background as shown in Fig. 2. The relatively low recall rate may be partly due to the inherent difficulty in distinguishing early caries lesions (D1-D2) on intraoral photographs. In addition, with a limited dataset, the model sometimes cannot recognize caries and classifies them as background. However, our calibration-based labeling procedure [17] aims to reduce variability and ensure high-quality ground truth annotations, thereby enhancing the model’s learning capacity despite real-world visual limitations.

In Fig. 2, we can observe that the model works very well for classes D0, D2, D5, and D6, where the correct predictions are 116, 109, 174, and 246, respectively. There is, however, great confusion in the background class, with a lot of cases from classes D0 to D6 being incorrectly classified as background, for example, 244 cases of D0 and 151 cases of D1. This shows that the model over-detects background, which may decrease sensitivity in early to advanced caries detection. There are also confusions between caries classes, for example, D3 being often classified as D2 or D4, and D4 being classified as D5 or background. In general, this confusion matrix shows that the model has great potential for certain categories but needs to be improved in terms of decreasing false negatives for caries and avoiding misclassifications to background.

**Fig. 3:**
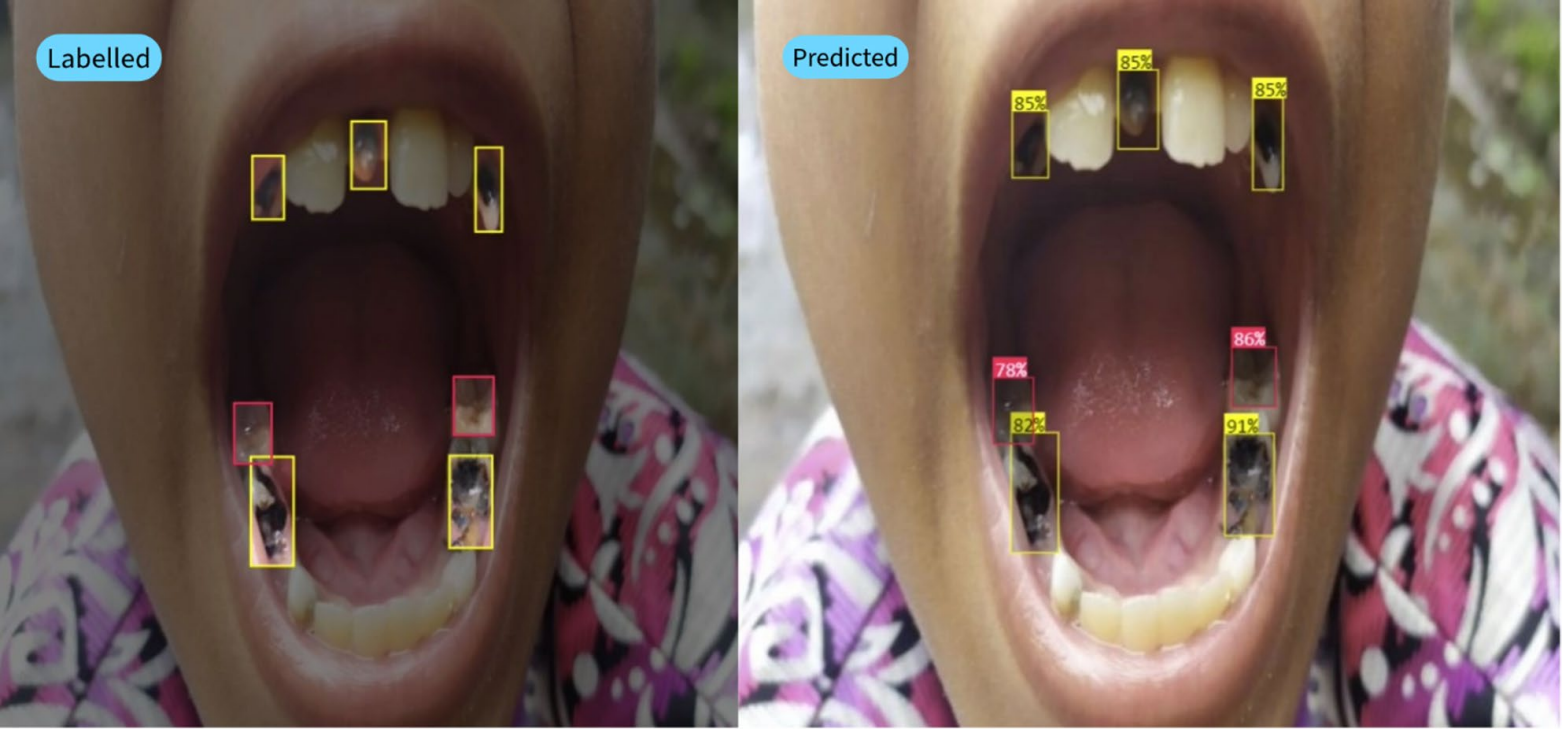
Analysis result on image with bounding box

This model was able to classify caries stages well, especially in the D5 and D6 classes. Late-stage caries are easier for doctors to classify than early stage caries are. In addition, the number of label instances in Table 1 shows that there are more data in the D5 and D6 classes, so the model learns more often in these classes, which contributes to the increased accuracy in assessing advanced caries. From the test results for the new data shown in Fig. 3, it was observed that the model predictions matched the manual labels, demonstrating the ability of the model to accurately detect caries. All predictions had confidence scores above 80%, reflecting the reliability of the model for recognizing caries with a high level of confidence. For example, the prediction of the right lower tooth had a confidence score of 91%, indicating that the model was very confident in its detection. This result indicates that the model was able to capture the caries pattern consistently according to the annotations made previously.

**Fig. 4:**
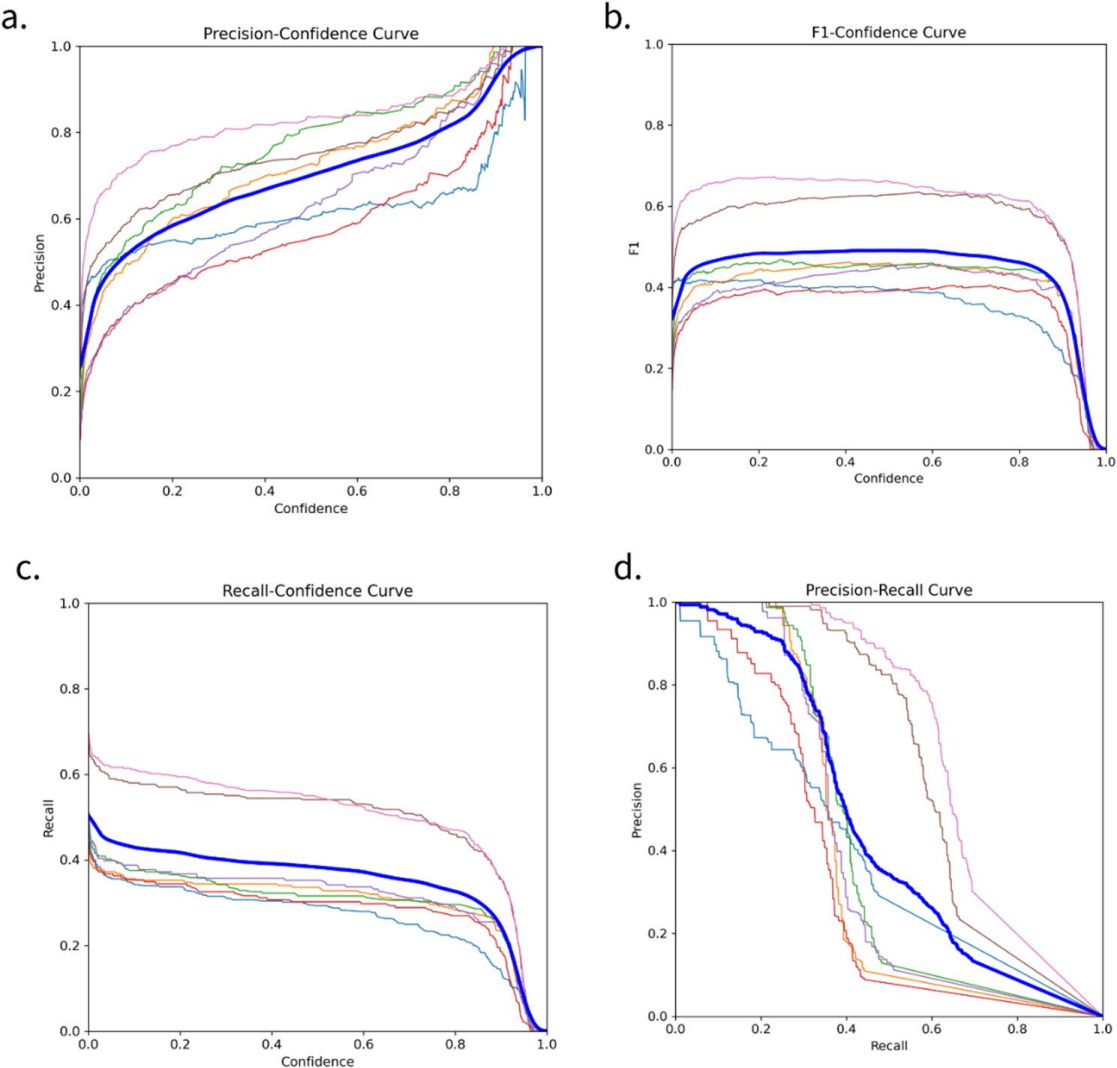
Performance graph of training results on YOLO-V8x

In Fig. 4a (precision - confidence curve), the increase in precision as confidence increases indicates the model’s ability to reduce false positives at higher confidence levels. A steep curve at low confidence reflects less accurate predictions; however, as confidence increases, the model becomes more confident and produces better precision. The stability of the performance at high confidence (close to 1) indicates that the model is effective in detecting clear data. However, the variation in the lower curve shows that some classes have varying precisions, indicating the model’s ability to detect certain classes better than others. In Fig. 4b (F1-Confidence Curve), the F1 score remains stable in the middle, but decreases at high confidence, indicating a trade-off between precision and recall. This is reinforced by Fig. 4c (Recall-Confidence Curve), where recall decreases as confidence increases, indicating that the model becomes more selective and misses some positive cases. Figure 4d (Precision-Recall Curve) emphasizes this trade-off, where an increase in recall is accompanied by a sharp decrease in precision. Although the model is effective in avoiding errors, the balance between correct positive detection and correct prediction results needs to be improved, especially in more complex or ambiguous classes.

After training the YOLO v8x model, a comparative test was conducted to assess the extent to which the results of the AI assessment were in line with those of dentists. This test used 320 intraoral photographs, in which the label frequency of each class varied. Of the 1,486 labels, class D1 had 201 labels (13.52%), whereas class D6 had the highest frequency with 277 labels (18.64%). Other classes, such as D2-D5, had a fairly even distribution, as shown in Table 4.

After conducting a comparative test between AI and dentist assessment, the results showed that there was no significant difference between the two methods in detecting and classifying caries at various stages of the ICDAS. Based on the data in Table 5, the P-value for each ICDAS class was greater than 0.05, indicating that the difference in results between AI and dentist assessment was not statistically significant. In line with the study, Moharrami et al. noted that various image processing techniques were used in the AI model, yet performance for different lesion severities remained consistent across studies, suggesting that AI can match dentists’ judgment in certain contexts [47]. This is consistent with findings from Devlin et al. who reported that AI systems served as an effective adjunct to traditional diagnostic methods, but the difference in diagnostic results was not statistically significant when compared to experienced dentists [48].

A comparison of the performance of AI and dentists in detecting caries in each ICDAS class from D1 to D6 shows that the difference between the two methods is not significant in line with the findings in the research of Shaya et al. [49]. In the early caries classes (D1 and D2), the sensitivity and specificity of AI were close to those of dentists, with P values greater than 0.05, indicating that AI was able to detect early stage caries with almost equal accuracy to dentists. In the D1 class, the sensitivity of AI (90.17%) was slightly higher than that of dentists (89.80%), whereas in the D2 class, AI had a slightly lower sensitivity (82.31%) than doctors (82.84%), but this difference was not statistically significant.

AI also performed competitively with high sensitivity and specificity in more advanced caries classes (D3-D6). At the D3 grade, the sensitivity of AI (83.12%) was very similar to that of dentists (83.53%). In classes D5 and D6, AI showed excellent performance, with a positive predictive value (PPV) almost identical to that of doctors, especially in class D5 (99.55% for AI and 99.43% for doctors). These results suggest that AI is superior in detecting late-stage caries, particularly on D5 and D6, which is supported by the availability of more data in these classes. Overall, AI provided comparable results to dentists in all ICDAS classes.

Apart from diagnostic equivalence, the AI model also showed a profound advantage in the speed of assessment. The results indicated that there was a significant comparison of caries assessment time. The average time generated by AI was 31.95 s with a standard deviation (SD) of 3.2 s, while the time taken by dentists to perform caries assessment was 133.61 s with a standard deviation of 6.53 s. This difference has a p-value of 0.000, indicating that the difference is statistically significant. These findings are consistent with those of previous studies that highlighted the efficiency of AI in diagnostic processes. For instance, Eschert et al. emphasized that AI can significantly reduce the time required for diagnostic assessments, allowing clinicians to allocate more time to patient care, which is crucial for enhancing patient satisfaction [50].

Based on these results, it can be concluded that AI can assist dentists in detecting caries faster than manual assessment by dentists. The speed generated by AI can provide benefits in terms of time efficiency, as well as the potential for increased accuracy in detecting caries at an early stage. This assertion is supported by Schropp’s research, which demonstrated that AI-assisted assessments in dental education led to quicker evaluations without compromising the diagnostic accuracy [51]. Furthermore, Güneç’s study indicated that AI tools could enhance decision-making under time constraints, thereby improving diagnostic performance in clinical settings [52]. The integration of AI into dental practice not only streamlines the assessment process but also holds promise for improving overall diagnostic outcomes, as noted by Khanagar et al., who reviewed the application of AI in the detection and diagnosis of dental caries [53].

**Fig. 5:**
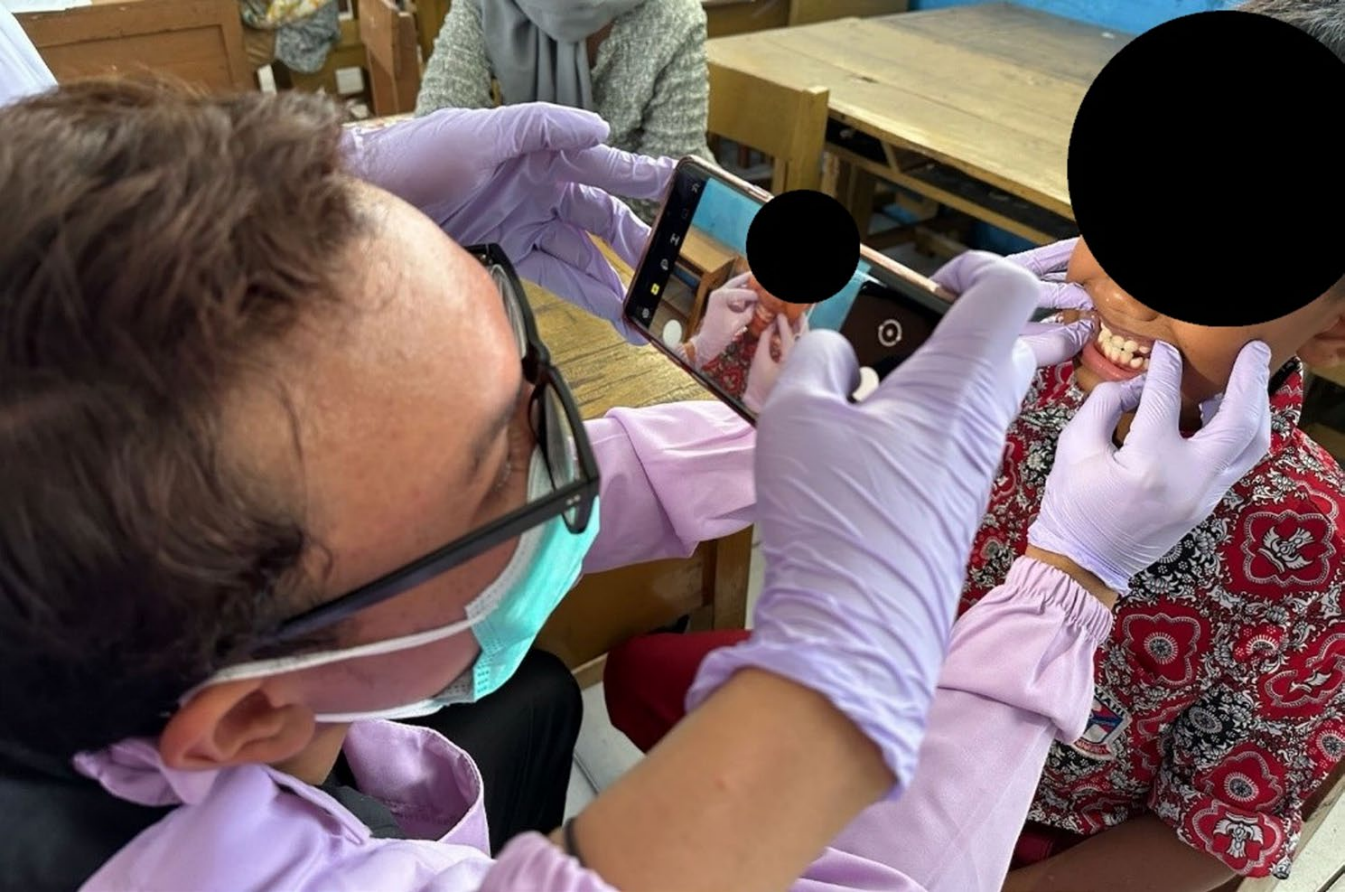
example of implementation for the UKGS program

Our research shows that the YOLO v8x deep learning model is capable of classifying caries effectively, as evidenced by various testing metrics, including sensitivity, specificity, and a comparative analysis of dental caries examination times between doctors and artificial intelligence. These results indicate that AI can significantly reduce the time required for caries assessment, which is a crucial factor in clinical practice. Future research could focus on doubling the datasets from all regions in Indonesia to create a more varied dataset for detecting caries stages. This approach aligns with findings from Alshaya et al., who noted that variations in data can influence the effectiveness of AI in clinical applications [54]. Moreover, expanding the dataset is essential for improving the robustness of AI models, as highlighted by Zhu et al., who found that a larger and more diverse dataset leads to improved diagnostic accuracy in dental radiographs [55]. In addition, the integration of artificial intelligence (AI) systems in this study can be incorporated into national school-based dental health programs such as UKGS, which can support large-scale early caries screening. This strategy can enhance the reach and efficiency of UKGS, as illustrated in Fig. 5, reduce reliance on limited dental health personnel, and accelerate the achievement of the national goal of caries-free children by 2030.

### Limitation

The limitation of this study was the position of the dental examination of the posterior part of the upper jaw, especially the second molar of the permanent teeth. With the help of tools such as intraoral mirrors, visibility can be improved. The operation of the AI system can be affected by the quality of the input image or photo and may vary. AI systems should be seen as a complementary tool that can improve diagnostic accuracy, not as a substitute for dentists in making a diagnosis.

In this study, we had a limited distribution of intraoral image datasets from D0 to D6, and the distribution in each class was imbalanced, but the differences were not too significant, as shown in Table 1. This may contribute to the overall decrease in accuracy because the AI model learns less from fewer labels than from more data. As a result, our study achieved a recall rate of 41.1%. To address this problem, we consider that deep learning algorithms can reduce the impact of such data. We conducted several experiments with deep learning algorithms, including Faster-RCNN, YOLO v8, and DETR, and obtained good results on YOLO V8x, which we present in this research paper [16].

## Conclusion

The YOLO-v8x Artificial Intelligence model, implemented in the HI Bogi Application for dental caries detection based on the ICDAS criteria (D1-D6), achieved sensitivity and specificity values exceeding 80%. This study demonstrated that the system performs comparably to dentists in the detection of dental caries. The application also proved to be highly efficient, completing examinations four times faster than the dentist’s assessment. Additionally, no differences were observed between the results of direct dental examinations and those obtained through HI Bogi application. These findings suggest that the HI Bogi AI-based system is an effective tool for detecting dental caries. However, the variation in precision between classes indicates that the model is inconsistent in detecting all classes with the same accuracy. This weakness indicates the need for improvements in handling complex classes and maintaining a balance between positive detection and prediction accuracy. Therefore, in future research, development could focus on using transformer-based AI models, which are known to be better at understanding complex patterns, to improve caries-detection accuracy. In addition, increasing the number of datasets with a wider variety will allow the model to learn more about caries categories, including lesions with unique characteristics that are rare.

## Data Availability

Data are available from the corresponding authors.
